# Preclinical evaluation of a commercially available biofilm disrupting wound lavage for musculoskeletal trauma

**DOI:** 10.1186/s13018-022-03199-x

**Published:** 2022-07-15

**Authors:** Michael E. Whitely, Sarah M. Helms, Preeti J. Muire, Alicia L. Lofgren, Rebecca A. Lopez, Joseph C. Wenke

**Affiliations:** Combat Wound Care Department, US Army Institute of Surgical Research, 3698 Chambers Pass, Building 3611, JBSA-Fort Sam Houston, San Antonio, TX 78234 USA

**Keywords:** Wound irrigation, Antibiofilm, Open fracture, Musculoskeletal infection

## Abstract

**Background:**

Treatment of open fractures remains a significant challenge in trauma care as these fractures are accompanied by extensive soft tissue damage, exposing the wound site to contaminants and increasing infection risk. Formation of biofilm, a capsule-like environment that acts as a barrier to treatment, is a primary mode by which infecting pathogens persist at the wound site. Therefore, a pressing need exists to identify irrigation methods that can disrupt biofilm and expose pathogens to treatment. This study aims to evaluate the antibiofilm wound lavage, Bactisure™, in comparison with saline for care of severe musculoskeletal wounds and elucidate potential effects on antibiotic treatment success.

**Methods:**

UAMS-1 *Staphylococcus aureus* biofilms were formed in vitro and treated with Bactisure™ wound lavage or sterile normal saline, alone, or in combination with sub-biofilm inhibitory levels of vancomycin. Characterization methods included quantification of biofilm biomass, quantification of viable biofilm bacteria, and biofilm matrix imaging. For in vivo assessment, a delayed treatment model of contaminated open fracture was used wherein a critical-sized defect was created in a rat femur and wound site inoculated with UAMS-1. Following a 6 h delay, wounds were debrided, irrigated with lavage of interest, and antibiotic treatments administered. Bacterial enumeration was performed on bone and hardware samples after two weeks.

**Results:**

An immediate reduction in biofilm biomass was observed in vitro following antibiofilm lavage treatment, with a subsequent 2- to 3- log reduction in viable bacteria achieved after 24 h. Furthermore, biofilms treated with antibiofilm lavage in combination with vancomycin exhibited a minor, but statistically significant, decrease in viable bacteria compared to irrigation alone. In vivo, a minor, not statistically significant, decrease in median bioburden was observed for the antibiofilm lavage compared to saline when used in combination with antibiotics. However, the percentage of bone and hardware samples with detectable bacteria was reduced from 50 to 38%.

**Conclusions:**

These results suggest that the antibiofilm wound lavage, Bactisure™, may hold promise in mitigating infection in contaminated musculoskeletal wounds and warrants further investigation. Here, we proposed multiple mechanisms in vitro by which this antibiofilm lavage may help mitigate infection, and demonstrate this treatment slightly outperforms saline in controlling bioburden in vivo.

## Introduction

Treatment of open fractures remain a significant challenge in trauma and military medicine. A 2007 examination by Owens et al. reported that approximately half of all U.S. personnel injured in Operations Iraqi Freedom and Enduring Freedom sustained open fracture injuries [1]. In addition to the damage sustained to the bone, these injuries are accompanied by severe damage to the surrounding soft tissue, exposing the wound site to environmental contaminants and increasing the likelihood of infection [2]. The current standard of care for open fractures includes surgical debridement of the injury site accompanied by irrigation of the wound space with sterile normal saline [3–6]. This is followed by administration of broad-spectrum antibiotics and treatment with appropriate fixation/regenerative medicine techniques. Despite this multi-tiered approach, infection rates in these injuries remain extremely high, with reported rates surpassing 25% in combat sustained tibial open fractures [7–9].

The precise bacterial burden that a wound must be reduced to in order to mitigate subsequent infection has not been clearly elucidated and is dependent on both wound environment and overall patient health [10–12]. As a result, the primary goal of debridement and irrigation procedures is to reduce bacterial burdens as much as possible while simultaneously removing non-viable tissue that may provide a substrate for colonization. Extended delays or suboptimal treatment approaches exacerbate infection by allowing infecting organisms adequate time to fully colonize the wound site and begin producing a protective matrix, termed biofilm. This capsule like environment provides protection from surveilling immune cells, a mechanical barrier to removal, and limits the local diffusion of antimicrobial therapies [13–15]. As a result, a pressing need exists to identify and optimize potential irrigation solutions that can actively disrupt biofilm, thereby reducing bacterial loads at the wound site and exposing the infecting organism to subsequent therapies.

Bactisure™ wound lavage is a commercially available, FDA approved antiseptic composed of ethanol, acetic acid, sodium acetate, benzalkonium chloride, and water and has been used in treatment of periprosthetic joint infections [16, 17]. Uniquely, this wound lavage acts to disrupt biofilm by deconstructing critical metallic linkages in the protective extracellular matrix and facilitates a high osmolarity environment that, coupled with a surfactant, promotes lysis of cells that have been exposed in their unprotected state. The aim of this study was to evaluate the ability of this antibiofilm irrigant (ABI) to restore susceptibility of biofilm bacteria to antibiotic treatment in vitro and investigate use in a preclinical model of severe musculoskeletal trauma. Many studies have reported on the presence of gram-positive bacteria in both acutely contaminated open fractures and recalcitrant infections, of which *Staphylococcus aureus* is a primary pathogen [7–9]. To this end, our group has implemented in vitro antibiofilm assays, and a clinically relevant model of contaminated open fracture utilizing a high biofilm-producing strain of *S. aureus* and delayed treatment [18, 19]. Outcomes in this preclinical model mirror those observed clinically, wherein there is only partial success at mitigating long term infection. Emergence of a readily available wound irrigation solution that can be easily incorporated into current standards of care, better minimize bacterial burdens in traumatic injuries, and maximize exposure to antibiotic therapies would prove invaluable.

## Materials and methods

### Biofilm formation

Biofilm was formed with UAMS-1 (ATCC 49230), a methicillin-susceptible osteomyelitis isolate of *S. aureus*, using a modified static incubation procedure previously detailed [20, 21]. Briefly, bacterial cultures were maintained at − 80 °C and sub-cultured on sheep’s blood agar plates overnight at 37 °C prior to experimental use. Fresh suspensions of UAMS-1 were prepared from overnight cultures and adjusted via optical density to a concentration of 5 × 10^6^ CFU/ml bacteria in cation-adjusted Mueller–Hinton Broth (MHB, Becton Dickinson, Franklin Lakes, NJ). Suspensions were supplemented with 2% human plasma and 200 μl aliquots inoculated in 96-well flat-bottomed polystyrene plates. Plates were incubated at 37 °C for 48 h to allow biofilm formation.

### In vitro biofilm dispersal and recovery of viable bacteria

Established biofilms were treated with a 2-min, static wash (200 μl) of Bactisure™ wound lavage (Zimmer Biomet) or sterile normal saline (0.9% sodium chloride, Baxter). Irrigant was removed and a second 2-min, static wash (200 μl) with normal saline was applied to both groups. This strategy was selected to reasonably replicate current clinical guidance where it is recommended that the lavage be used at the end of procedure prior to wound closure, and followed shortly by secondary irrigation with saline [16]. Samples indicated for additional incubation received 200 μl of fresh MHB media or MHB media supplemented with 32 μg/mL of vancomycin and were incubated for an additional 24 h at 37 °C. Following incubation, biofilms were gently washed with normal saline and biomass determined by staining with 100 μl of 0.1% Crystal Violet (Sigma-Aldrich, St. Louis, MO) for 30 min at room temperature. Excess stain was removed, biomass stain solubilized in 95% ethanol, and biomass quantified by measuring OD of supernatant at 570 nm (Cytation 5, BioTek). To determine viability of biofilm bacteria, incubated biofilms were gently washed and biofilm bacteria removed by sonication for 15 min. Viable colony forming units (CFUs) were quantified by enumerating serial dilutions on blood agar plates (Remel, Lenexa, KS) following bacteria removal. All assays were performed in triplicate.

### Confocal scanning laser microscopy

Biofilms were visualized by staining with Film Tracer™SYPRO^®^ Ruby Biofilm Matrix Stain (Invitrogen) and imaging with a confocal laser scanning microscope (Zeiss 900, Zeiss). Briefly, bacterial suspensions were prepared and supplemented with 2% human plasma as outlined above. Suspensions (2 ml) were inoculated in 6-well glass bottom plates and incubated at 37 °C for 48 h. Established biofilms were treated with a 2-min, static wash (2 ml) of Bactisure™ wound lavage or sterile normal saline as outlined above. Irrigant was removed and a second 2-min, static wash (2 ml) with normal saline was applied to both groups. Samples indicated for additional incubation received 2 ml of fresh MHB media and were incubated for an additional 24 h at 37 °C. Following treatment, biofilms were gently washed with normal saline, fixed with 4% paraformaldehyde (10 min), and stained with Film Tracer™SYPRO^®^ Ruby Biofilm Matrix Stain according to manufacturer’s instructions (30 min). Matrix-stained *Z* stack images were acquired at 20× magnification using Texas red^®^ filter. At least five images were taken from distinct regions within the glass area of the wells, and representative images were selected for each treatment group. The assay was performed in triplicate.

### Animals

Research was conducted in compliance with the Animal Welfare Act, the implementing Animal Welfare regulations, and the principles of the Guide for the Care and Use of Laboratory Animals, National Research Council. The facility’s Institutional Animal Care and Use Committee approved all research conducted in this study. The facility where this research was conducted is fully accredited by the AAALAC. Animals were assigned to experimental groups where they received saline or antibiofilm lavage-based wound irrigation followed by systemic or systemic plus local antibiotic treatments, Table 1. The Saline (−) control group was kept to a reduced animal number as overwhelming infection was observed in all animals. Male Lewis rats (325–400 g; ~ 11 weeks of age) received pre-surgical administration of buprenorphine‐SR (1.2 mg/kg; s.c., ~ 30 min prior) for pain management and were observed post-surgery for signs of distress and abnormal changes in mobility. Animals were euthanized under anesthesia after 2 weeks with a lethal dose of pentobarbital (Fatal Plus) and tissues and fixation hardware harvested.

**Table 1:** Experimental groups

Treatment group	Sample size	Irrigation solution	Systemic treatment	Local treatment
Saline (−)	4	Normal Saline	Cefazolin (5 mg/kg)	–
Saline (+)	16	Normal Saline	Cefazolin (5 mg/kg)	Vancomycin (50 mg)
ABI (+)	16	Bactisure™ Wound Lavage	Cefazolin (5 mg/kg)	Vancomycin (50 mg)

### Contaminated open fracture model

**Fig. 1:**
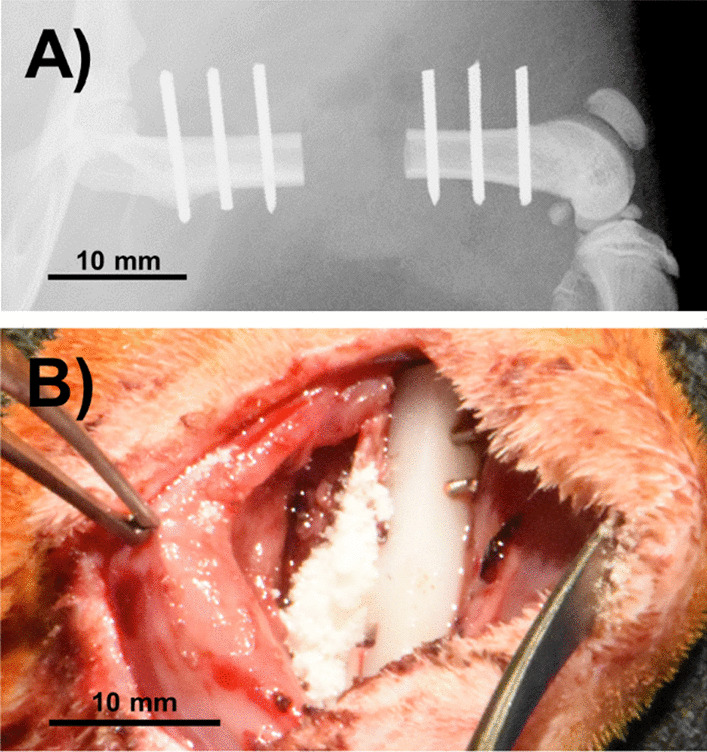
**A** Radiographic representation of the plate and wire placement in a 6 mm defect model. **B** Placement of the locally applied vancomycin powder into and immediately surrounding the 6 mm defect in the femur. Scale bar equal to 10 mm

A contaminated, critical-sized defect was created in the in the mid-diaphysis of the femur as previously described [18]. Briefly, animals were anesthetized and prepped for surgery under aseptic conditions. A lateral incision was made to expose the anterolateral aspect of the femoral shaft, and periosteum and adjacent muscle were stripped from the bone. A radiolucent plate was fixed on the anterolateral surface of the femur using six 0.9 mm diameter threaded Kirschner wires. A 6 mm segment of bone was removed using a reciprocating saw and the defect packed with 100 mg sterile collagen wetted with 10^5^ CFU of UAMS-1 *S. aureus* (ATCC 49230) in sterile saline. The wound was closed and animal allowed to recover for six hours. Correct pin and plate placement was confirmed via X-ray analysis, Fig. 1A. Following six-hour incubation, animals were anesthetized, wounds debrided of collagen and non-viable soft tissue, and low-pressure irrigation applied. Animals assigned to saline irrigation groups received an irrigation volume of 60 ml. Animals assigned to the biofilm disrupting wound lavage group received 2 ml Bactisure™ Wound Lavage, a volume sufficient to fill the wound space, followed by secondary irrigation with 10 ml of saline 2 min later. This strategy was selected to reasonably replicate current clinical guidance where it is recommended that the lavage be used at the end of procedure prior to wound closure, and followed shortly by secondary irrigation with saline [16]. Animals assigned to groups receiving local antibiotic treatment, Saline (+) and ABI (+), received 50 mg vancomycin powder placed within the defect and wound pocket, Fig. 1B. The wound was then closed with suture and skin clips and the animal allowed to recover. To replicate clinical protocol, all animals received twice daily broad-spectrum systemic antibiotic (cefazolin; SQ, 5 mg/kg) for 72 h following surgery.

### Bacteria bioburden enumeration

Following aseptic harvesting of the hind limb, fixation hardware was removed from the femur and samples prepared for processing. Fixation hardware was suspended in normal saline and sonicated to remove bacteria from the implant surface. Femurs were flash-frozen in liquid nitrogen, crushed to a fine powder, suspended in normal saline, and vortexed. Bioburden was quantified by plating serial dilutions of the bone and hardware samples onto blood agar plates (Remel, Lenexa, KS). Plates were incubated overnight at 37 °C and colony forming units counted and normalized to weight of the explanted material.

### Statistics

Statistical analysis was conducted using GraphPad Prism 7.01 (GraphPad Software Inc., La Jolla, CA). In vitro data presented as mean ± standard deviation with statistical significance defined as *p* < 0.05 using Student’s *t*-test. In vivo CFU values represented as median ± interquartile range with statistical significance defined as *p* < 0.05 using Kruskal–Wallis test with Dunn's multiple comparison test. Fisher's exact test was performed to compare the rate of samples with detectable bacteria (detection identified as 30 CFU per gram).

## Results

### In vitro biofilm dispersal and bacteria viability

**Fig. 2:**
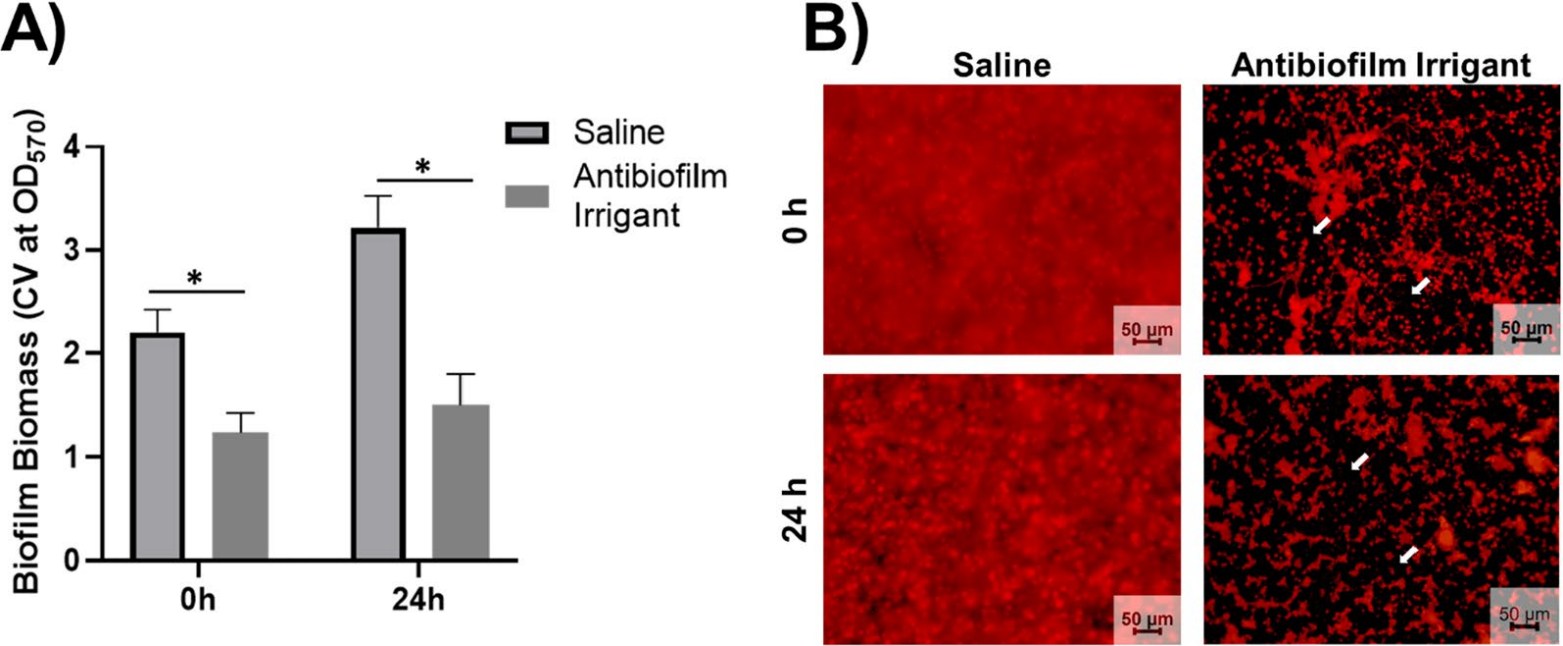
**A** Assessment of irrigant mediated biofilm dispersal determined by quantification of absorbance of solubilized crystal violet from stained biofilms 0 and 24 h after treatment. **B** Representative CLSM images of biofilm dispersal and inhibitory activity 0 and 24 h after irrigation. Arrows indicate representative areas of biofilm removal (no staining) following irrigation. Mean ± standard deviation represented, and significance determined between groups using Student’s *t*-test, *p < 0.05

**Fig. 3:**
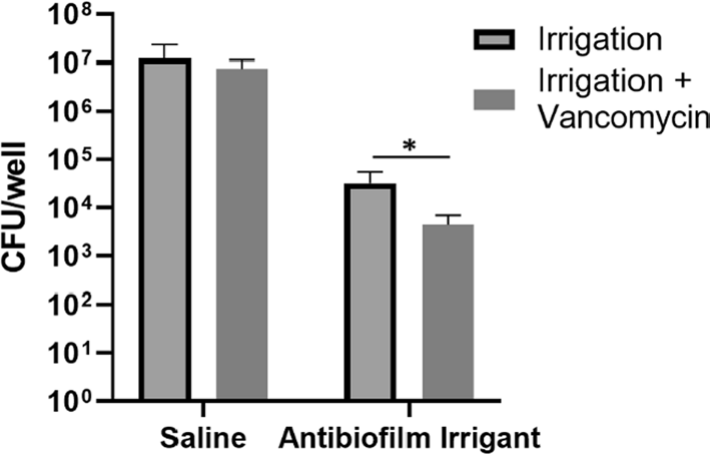
Irrigant mediated effects on biofilm bacteria viability 24 h after irrigation, with and without subsequent vancomycin treatment. Mean ± standard deviation represented, and significance determined between groups using Student’s *t*-test, *p < 0.05

The ability of the antibiofilm irrigant to provide rapid disruption and dispersal of established biofilm was determined by quantifying total biofilm biomass immediately following irrigation. An approximate 43% decrease in biofilm biomass was identified for the ABI compared to saline control, with a 53% decrease present after 24 h, Fig. 2A. Dispersal of biofilm was further confirmed via imaging of the biofilm matrix immediately and 24 h after irrigation, Fig. 2B. Additionally, irrigation with the ABI yielded a 2- to 3- log reduction in viable bacteria after 24 h compared to saline irrigation, Fig. 3. ABI samples further treated with 32 µg/ml vancomycin exhibited an added minor (0.5–1.0 log), but statistically significant (*p* < 0.05) decrease in viable bacteria compared to irrigation alone, Fig. 3. In contrast, saline irrigated samples did not exhibit a significant difference in viable bacteria after application of vancomycin treatment.

### Performance in contaminated open fracture model

**Fig. 4:**
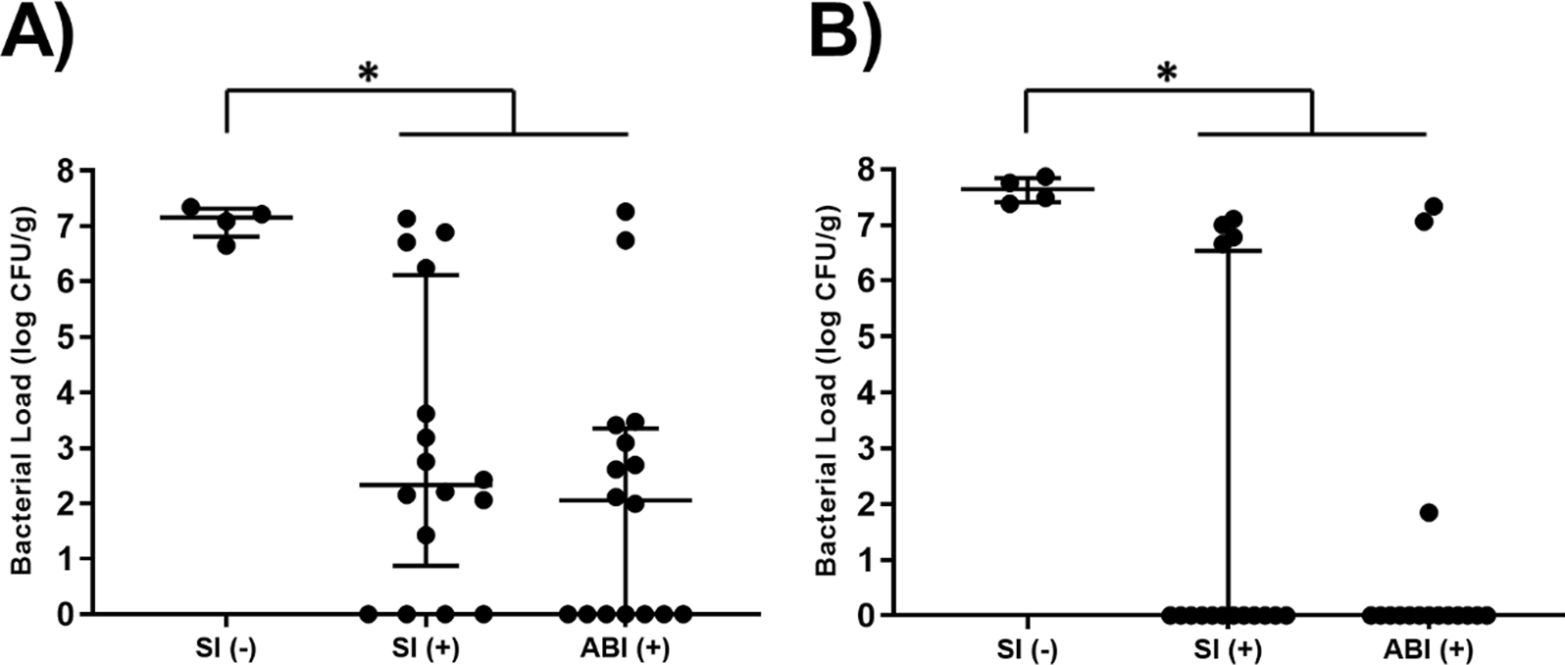
**A** Bacterial enumeration of *S. aureus* (UAMS-1) in bone tissue (log CFU/g). **B** Bacterial enumeration of *S. aureus* (UAMS-1) on removed hardware implant (log CFU/g). Median ± interquartile range represented, and significance determined between groups using Kruskall-Wallis test followed by Dunn's multiple comparisons test, *p < 0.05. Group descriptions: SI (−): Saline irrigant; SI (+): Saline irrigant plus local antibiotic; ABI (+): Antibiofilm irrigant plus local antibiotic. All groups received systemic antibiotic

All animals in the Saline (−) control group that received saline-based irrigation and systemic administration of broad spectrum antibiotics, without the addition of local treatment at the wound site, developed robust infection after 14 days with a median bacterial load of 1.4 × 10^7^ CFU/g bone tissue, Fig. 4. Addition of local vancomycin treatment to saline-based irrigation procedures resulted in a > 4 log reduction in median bone bioburden compared to the Saline (−) control group. However, 75% of bone samples still retained detectable levels of bacteria, Table 2. Substitution of saline irrigation with the antibiofilm irrigant in the combination (systemic plus local antibiotic) treatment approach yielded only a marginal improvement in median CFU levels, Fig. 4. Despite this, the number of bone samples with detectable bacteria was reduced to 56%. With respect to fixation hardware, both irrigation groups that included local antibiotic treatment resulted in total eradication of bacteria from a minimum of 75% of samples. Combined, the percentage of bone and hardware samples with detectable levels of bacteria was reduced from 50% for Saline (+) treatment group to 38% for ABI (+) treatment group, Tables 2 and 3.

**Table 2:** Proportions of samples with detectable bacteria

Treatment group	Detectable bone samples (% total)	Detectable hardware samples (% total)
Saline (−)	4 (100)	4 (100)
Saline (+)	12 (75)	4 (25)
ABI (+)	9 (56)	3 (18)

**Table 3:** Similarity of the effect of treatment on rate of bacteria detection

Treatment group	*P* of comparison with saline (−) (bone samples)	*P* of comparison with saline (−) (hardware samples)
Saline (+)	0.54	0.03
ABI (+)	0.25	0.01

*P* generated by Fisher exact test

## Discussion

The aim of this study was to determine whether irrigation of a contaminated, open fracture injury with the antibiofilm wound lavage, Bactisure™, would render a high biofilm-producing strain of *S. aureus* more susceptible to antibiotic treatment and reduce bioburden compared to saline irrigation. Formation of biofilm, a protective, heterogeneous matrix of polysaccharides, proteins, and nucleic acids, is a primary mode by which pathogens persist at the wound site [22]. Physical barriers to clearance and drug diffusion provided by the matrix are accompanied by cellular changes in gene expression and metabolic activity, all serving to reduce susceptibility to antibiotic treatment [22, 23]. As a result, biofilm mitigation is a primary factor in determining treatment outcome. Debridement and irrigation remain a first-line defense to reduce bioburdens and mitigate infection, however, a lack of consensus remains on the most optimal irrigation practice [24, 25]. Although surfactant- and antibiotic-based alternatives have been extensively explored, these options often fail to deliver on promising in vitro activity and have yet to overtake saline irrigation as standard of care. Highlighting this point, a multi-center clinical trial recently reported that irrigation of extremity open fractures with castile soap, a commonly selected alternative, served to increase infection rate, from 11.6% with saline use, to 14.8% with use of castile soap [26].

Recently, multiple studies have demonstrated Bactisure™ mediated antibiofilm activity on orthopaedic materials common in joint replacement including cobalt-chrome, titanium, stainless steel, and polymethylmethacrylate [27–29]. To further investigate the potential of this technology in other areas of orthopaedic practice, an in vitro assessment of antibiofilm activity in combination with antibiotic treatment was performed, followed by an evaluation of efficacy in a rat model of contaminated musculoskeletal trauma. Three distinct effects were identified in vitro that may ultimately contribute to mitigation of infection in contaminated musculoskeletal wounds. These results illustrate direct effects of irrigation on overall removal of biofilm, as well as secondary effects that may improve efficacy of subsequent treatments. First, an immediate removal of biofilm was achieved as evidenced by a 43% decrease in remaining biomass immediately following irrigation with the ABI compared to saline. This antibiofilm irrigant acts to disrupt established biofilm by using a combination of buffers and physiologic acids to produce a chelating environment and competitively bind metallic bonds holding the biofilm matrix together, thereby releasing the polymeric substrates and dissolving the matrix environment [17]. Second, a reduction in viable colony forming units was observed after irrigation, suggesting the combination of buffers and surfactants present in the lavage create an environment toxic to contaminating pathogens. Finally, and most interestingly, a partial restoration of antibiotic mediated inhibitory activity was observed. This in vitro study employed vancomycin treatment at a concentration below established biofilm inhibitory levels [21]. The observed decrease in viable bacteria with vancomycin treatment after irrigation with the ABI suggests dispersal of the surrounding matrix is successfully exposing unprotected cells to local treatments, a phenomenon not observed following irrigation with saline. This is highly promising as it has been reported that, although *S. aureus* biofilms can be eradicated from orthopaedic implants using vancomycin alone, the time and concentration profiles needed to achieve this are not sustainable with current systemic or local delivery platforms [30]. Although wound care products similar in formulation to the antibiofilm irrigant studied here have successfully eliminated superficial wound infections of *S. aureus* and *Pseudomonas aeruginosa*, the ability of these technologies to mitigate infection in preclinical models of significant musculoskeletal injury has remained unknown [31].

We have previously demonstrated that the efficacy of both wound irrigation and antibiotic treatment significantly diminishes as the time delay between contamination and treatment increases [32, 33]. In the present study, it was observed that debridement and saline irrigation six hours after contamination, followed by systemic and local antibiotic treatment, resulted in 50% of total bone and hardware samples containing detectable bacteria. When saline irrigation was replaced with irrigation with the biofilm disrupting wound lavage, a 25% reduction in total samples with detectable bacteria was observed. Although this preliminary assessment identified only a minor, not statistically significant decrease in median bioburden, it is noteworthy that a rebound in bacterial load was not observed. In a goat model of musculoskeletal wound infection, bioburden rebounds for castile soap, benzalkonioum chloride, and bacitracin irrigants all exceeded saline irrigation [34]. Furthermore, chlorohexidine gluconate-based irrigation solutions have previously failed to reduce bioburden levels in the rodent model implemented here, and authors of that study cautioned against potential secondary damage to the wound site [19].

Although a significant reduction in bioburden was not observed, a decrease in overall number of samples with detectable bacteria suggests possible clinical relevance in these injuries and potential for further optimization. Irrigation volume and wound contact time present as variables that can be easily manipulated to optimize efficacy, and it should be noted that the current study employed only a single application strategy that models clinical use in periprosthetic joint infections [16]. It is none the less encouraging that these results were achieved using 20% volume compared to saline irrigation. This holds particular relevance to use in austere, prolonged field care scenarios where space and weight restrictions are implemented, yet clinical guidelines recommend repeated irrigation of injury sites [35].

## Conclusions

This study serves as an additional data point to suggest that use of the biofilm disrupting wound lavage, Bactisure™, may hold promise in the care of severe musculoskeletal wounds and warrants further investigation. Here, we proposed multiple mechanisms by which this antibiofilm irrigant may help mitigate infection and demonstrate this technology slightly outperforms saline in helping reduce bioburden in a delayed treatment model of contaminated open fracture. Ultimately, this wound lavage may prove a useful tool in the design of novel treatment paradigms to mitigate infection in severe musculoskeletal injuries.

## Data Availability

The datasets used and/or analyzed during the current study are primarily presented in the current manuscript and are available from the corresponding author on reasonable request.
